# The Fundamentals of Halide Perovskite Instability: CsPbBr_3_ as a Model System

**DOI:** 10.3390/nano16191264

**Published:** 2026-10-07

**Authors:** Neena Prasad, Achiad Goldreich, Alen Sam Thomas, Philip Nathaniel Immanuel, Lena Yadgarov

**Affiliations:** Department of Chemical Engineering, Ariel University, Ariel 4070000, Israel; neenaphy@gmail.com (N.P.); achiod@ariel.ac.il (A.G.); alensam111@gmail.com (A.S.T.)

**Keywords:** halide perovskites, instability, ion migration, defect formation, anharmonicity

## Abstract

Halide perovskites (HPs) exhibit outstanding optoelectronic properties, making them highly attractive for advanced photonic and electronic applications. Among them, cesium lead bromide (CsPbBr_3_) exhibits high photoluminescence efficiency, narrow emission linewidths, and excellent color purity, making it suitable for light-emitting diodes, photodetectors, scintillators, and lasers. However, its practical application remains limited by intrinsic instability and environmental stressors such as moisture, heat, oxygen, and illumination. This review discusses the fundamental degradation mechanisms in HPs, using CsPbBr_3_ as a representative system, primarily in nanocrystals (NCs), while also considering thin-film and bulk forms. Intrinsic instability arises from the soft ionic lattice, a relatively low tolerance factor, and anharmonic lattice dynamics, which promote defect formation, ion migration, and structural distortion. Low activation energies for ionic transport enable migration of halide (Br^−^) and A-site (Cs^+^) ions, leading to defect accumulation, non-radiative recombination, and phase instability. In NCs, surface-dominated effects further accelerate degradation, driven by high defect densities and ligand desorption. Extrinsic factors promote degradation through hydrolysis, photoinduced defect formation, radical-driven reactions, and transformation into secondary phases. These processes can be intensified when multiple environmental stressors act simultaneously, reducing operational stability. These processes form a coupled, condition-dependent degradation network. Their interactions and relative contributions depend on the material form and environmental or operational conditions. Overall, this review highlights the interconnected roles of lattice dynamics, defect formation, ionic transport, and environmental interactions in governing the instability of CsPbBr_3_-based HPs.

## 1. Introduction

Perovskites represent a broad class of crystalline materials with the general formula ABX_3_. Their structure consists of a three-dimensional network of corner-sharing BX_6_ octahedra, where A is a monovalent cation, B is a divalent metal cation, and X is an anion [1,2]. Perovskites exhibit a wide range of electrical, optical, and structural properties depending on the chemical composition. This versatility enables applications in photovoltaics, catalysis, sensing, and optoelectronics [3,4,5,6,7]. Among these materials, halide perovskites (HPs), where X is a halide anion (Cl^−^, Br^−^, or I^−^), have gained considerable attention due to their excellent optoelectronic properties. All-inorganic HPs, particularly cesium lead halides such as CsPbX_3_ (X = Cl^−^, Br^−^, I^−^), exhibit tunable band gaps, high absorption coefficients, high photoluminescence quantum yields (PLQY), and narrow emission linewidths [8,9,10]. These characteristics make them highly attractive for light-emitting diodes (LEDs), low-threshold lasers, photodetectors, radiation detectors, and related optoelectronic devices [11,12,13,14]. Within this family, CsPbBr_3_ has emerged as a model system due to its comparatively higher resistance to moisture and oxidation relative to hybrid organic–inorganic or iodide-based perovskites [15,16]. Despite these advantages, CsPbBr_3_ remains sensitive to thermal stress, illumination, and exposure to polar solvents. These conditions induce phase transitions, defect formation, and structural degradation, ultimately limiting device stability and practical applicability [17].

The instability of HPs originates from both intrinsic material properties and external environmental stressors. Their soft ionic lattice and defect-prone surfaces and interfaces make these materials susceptible to structural and chemical changes [18,19,20,21,22]. External factors such as temperature, illumination, electric fields, moisture, and polar solvents further accelerate degradation through lattice strain, surface reactions, and chemical decomposition [23,24,25,26]. The extent of these effects strongly depends on material architecture, with NCs and thin films being particularly sensitive because of their high surface area and numerous interfaces and grain boundaries [27,28,29].

In CsPbBr_3_, instability is closely associated with its dynamically soft Pb–Br framework and structural distortions of the PbBr_6_ octahedra [30,31]. These characteristics facilitate ionic motion and become particularly important at surfaces and grain boundaries, where structural disorder and defect-rich regions can accelerate degradation [26,31,32,33]. External stressors such as illumination, temperature, electric fields, and polar environments further amplify these processes by promoting ion redistribution and chemical degradation [34,35,36]. These characteristics provide the basis for examining the intrinsic and extrinsic origins of instability in CsPbBr_3_.

Several reviews have examined the stability of HPs from different perspectives, including defect-mediated degradation, ion migration, environmental stability, and mitigation strategies [37,38,39]. However, these studies generally address broad classes of perovskite compositions or focus on specific degradation mechanisms and device architectures. Consequently, the relationships among lattice dynamics, ionic transport, defect evolution, and degradation within a single representative inorganic perovskite remain less systematically integrated. In this review, CsPbBr_3_ is used as a model system to critically examine these relationships across NCs, thin films, bulk crystals, and device architectures. Rather than treating individual degradation mechanisms as independent or proposing a universal degradation sequence, the available evidence is evaluated within a coupled, condition-dependent framework. Particular attention is given to experimental observations, experimentally inferred relationships, and theoretical predictions. Differences arising from material form, length scale, and experimental or operational conditions are also considered. This approach allows established relationships to be distinguished from those that remain insufficiently resolved. The distinctive contribution of this review is the integration of intrinsic lattice characteristics, defect chemistry, ionic transport, and external stressors while distinguishing intrinsic instability from extrinsic degradation and emphasizing their coupling under realistic conditions.

Understanding HP instability requires a systematic analysis of its underlying physical and chemical mechanisms. Since synthesis conditions strongly influence crystal structure, morphology, surface chemistry, and defect populations, the major preparation strategies for CsPbBr_3_ are first discussed. The subsequent sections examine the intrinsic factors governing instability, including lattice geometry, anharmonic lattice dynamics, ion migration, defect formation, and phase instability. This is followed by an assessment of extrinsic degradation induced by moisture, oxygen, illumination, temperature, electric fields, and polar solvents. Finally, the interplay among these degradation processes is considered to establish how intrinsic material properties and external stressors collectively determine the stability of CsPbBr_3_-based systems. This review summarizes previously published experimental and theoretical studies on CsPbBr_3_ instability; no original unpublished experimental data are presented.

## 2. Synthesis

**Figure 1:**
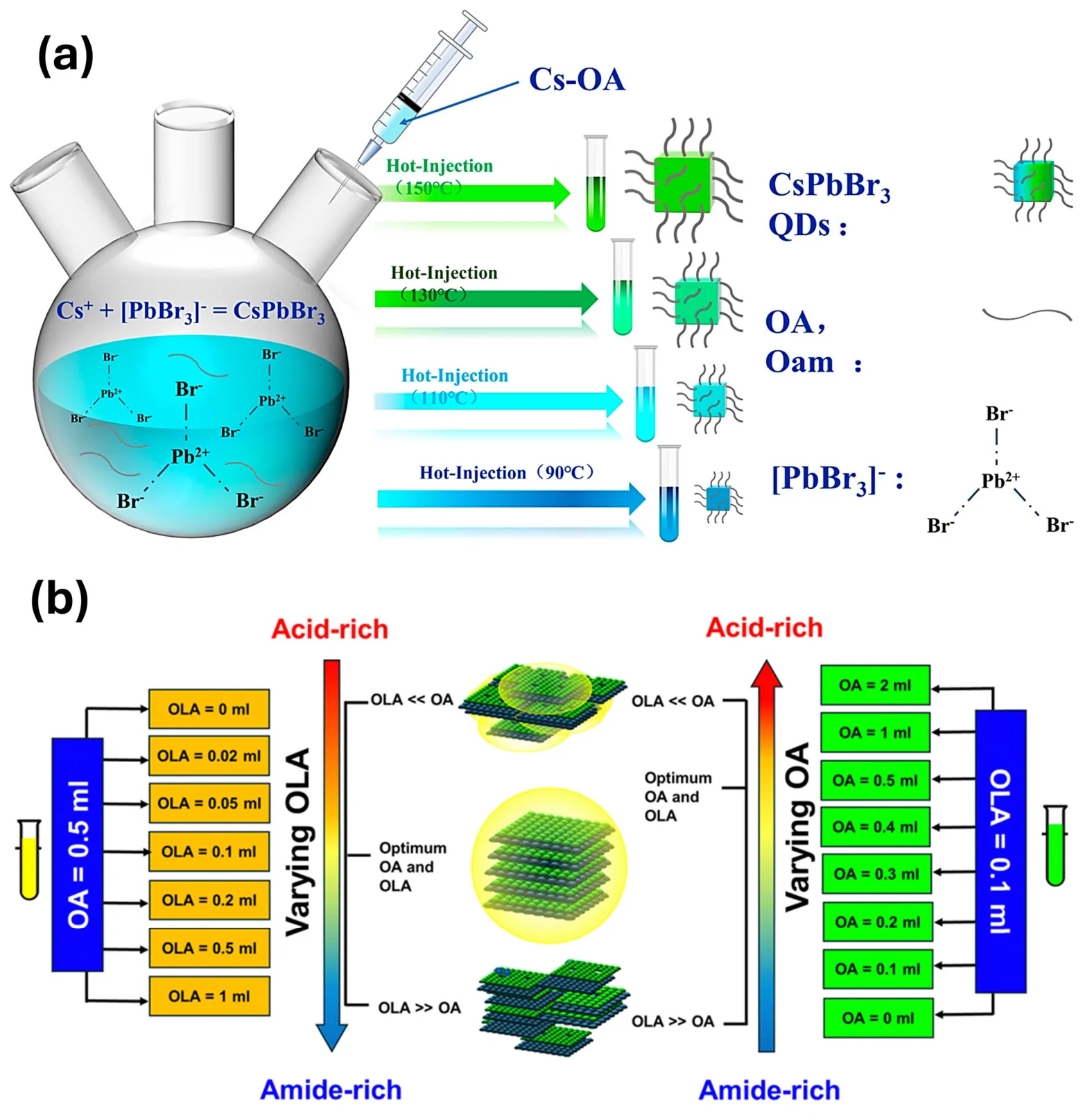
Schematic illustrations of (**a**) the hot-injection (HI) synthesis of CsPbBr_3_ NCs [54] and (**b**) oleic acid (OA)/oleylamine (OAm)-controlled growth of CsPbBr_3_ nanostructures [55]. Reproduced with permission from the respective references.

The synthesis of HP NCs plays a critical role in influencing their structural quality, defect density, and long-term stability. Therefore, various synthesis strategies have been developed to control composition, morphology, and optical properties. These methods are generally classified into two categories: wet synthesis or solution-based synthesis [40,41,42,43] and dry synthesis, or solid-state synthesis [44]. The solution-based synthesis of CsPbBr_3_ NCs primarily includes the hot-injection (HI) method, ligand-assisted reprecipitation (LARP), and solvothermal synthesis. Among these approaches, the HI method has served as a benchmark route for producing high-quality CsPbBr_3_ NCs with precise control over size, morphology, and composition while yielding high PLQY [3]. In HI synthesis, ligand chemistry critically controls the growth of CsPbBr_3_ NCs. Strongly bound carboxylate ligands favor the formation of larger isotropic nanocubes. In contrast, weakly bound alkylammonium ligands promote thinner, anisotropic nanoplatelets and are readily displaced by polar solvents. Reaction temperature further controls morphology, with lower temperatures favoring quasi-2D nanoplatelets and higher temperatures yielding nanocubes (Figure 1a) [45]. In contrast, LARP offers a simple, room-temperature alternative that enables rapid synthesis and facile compositional tuning, although it generally exhibits lower reproducibility and long-term stability (Figure 1b) [46,47]. Both methods highlight the critical roles of surface ligands, reaction temperature, and quantum confinement in governing the optical properties of CsPbBr_3_ NCs. Despite their success at the laboratory scale, challenges related to weak ligand binding, long-term stability, and scalability persist. These limitations motivate the development of alternative synthesis strategies, which are discussed below.

Solvothermal synthesis is a widely used solution-based approach for the preparation of HP NCs. It enables controlled nucleation and crystal growth under elevated temperature and pressure, yielding highly crystalline materials with tunable optical properties. In addition to solvothermal synthesis, emulsion, ultrasonication, and microwave-assisted methods provide efficient solution-based routes for HP NCs. Emulsion synthesis confines nucleation within microdroplets formed by immiscible solvents and surfactants, enabling the formation of highly uniform CsPbX_3_ quantum dots (QDs) with high PLQY and good colloidal stability [24]. Recent developments have focused on improving scalability and extending this approach to mixed-halide and heterostructured perovskite NCs for broader optoelectronic applications [48]. Ultrasonication uses acoustic cavitation to accelerate precursor dissolution and nucleation, enabling rapid and scalable preparation of composition-tunable APbX_3_ NCs with promising optical performance [49]. Microwave-assisted synthesis offers fast, reproducible, and energy-efficient crystallization with wide emission tunability and growing potential for doped materials and continuous-flow, large-scale production [50].

Mechanochemical synthesis offers a rapid, solvent-free route to HP NCs through ball-milling of stoichiometric halide salts and was systematically demonstrated for CsPbBr_3_ in 2019 [51]. Short milling times (≤5 min) yield mixed ternary phases, including CsPbBr_3_ and Cs_4_PbBr_6_, accompanied by dual photoluminescence (PL) features. In contrast, prolonged milling leads to particle fragmentation and defect formation, resulting in reduced emission intensity due to mechanically induced disorder [51]. Guided mechanochemical strategies have enabled the formation of host–guest heterostructures, such as Cs_4_PbBr_6_/CsPbBr_3_ endotaxial composites. These composites significantly enhance PLQY and stability by confining the emissive CsPbBr_3_ domains within a stabilizing Cs_4_PbBr_6_ matrix [52]. More recent developments incorporating surfactants or wet-milling conditions have improved colloidal dispersibility, PL efficiency, and enabled access to lead-free perovskite compositions [53].

High-temperature solid-state synthesis is a foundational method for producing phase-pure bulk HPs and establishing thermodynamic phase relationships among CsPbX_3_, Cs_4_PbX_6_, and CsPb_2_X_5_ compounds [56]. Although this approach does not directly yield NCs, subsequent quenching, grinding, or exfoliation steps have enabled access to nanoscale domains and excitonic behavior. This method has also been extended to lead-free systems such as Cs_2_AgBiBr_6_ [57]. As a complementary high-temperature strategy, molten salt synthesis utilizes molten inorganic salts as reactive solvents that promote homogeneous nucleation, enhanced ion diffusion, and high crystallinity. This approach yields CsPbBr_3_ NCs with narrow emission linewidths, high PLQY, and improved thermal stability, offering a scalable, environmentally benign route to high-quality perovskite NCs [58,59]. The synthesis-dependent structural and optical characteristics of CsPbBr_3_ prepared through various approaches are summarized in Table 1. Stability parameters are not directly compared because the reported testing conditions, durations, and evaluation criteria vary substantially among studies.

**Figure 2:**
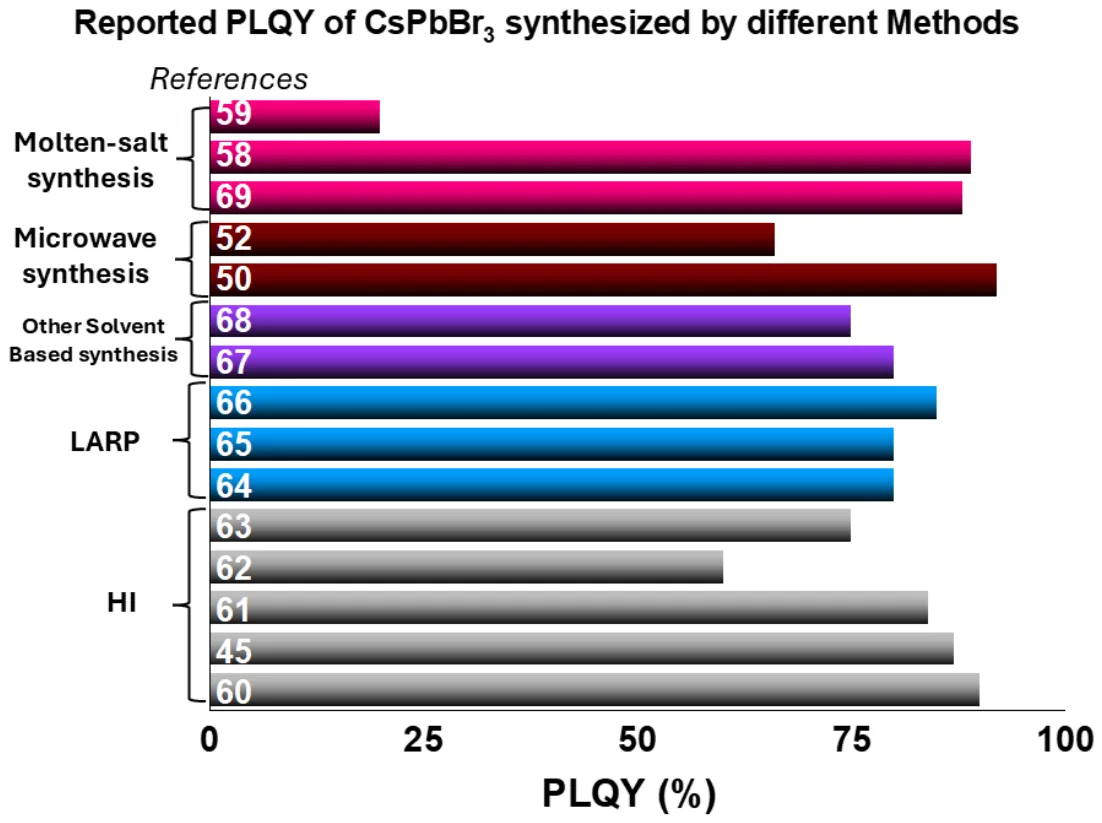
Comparison of literature-reported PLQY values for CsPbBr_3_ prepared using different synthesis approaches. Values were obtained from independent studies under different sample and measurement conditions; therefore, direct quantitative comparison should be made with caution. Uniform error bars are not shown because study-specific uncertainties were not consistently reported.

Although these synthesis methods provide precise control over the composition, morphology, and optical properties of CsPbBr_3_, they also strongly influence crystal quality, defect density, surface chemistry, and ligand binding. Since these factors govern ion migration, defect formation, and environmental susceptibility, synthesis plays a critical role in determining the long-term stability of CsPbBr_3_. Therefore, understanding instability requires consideration of both the intrinsic characteristics of the perovskite lattice and the structural features introduced during synthesis. PLQY can serve as an indirect indicator of defect passivation because non-radiative recombination is strongly influenced by defect states. Among the reported synthesis routes, solution-based methods such as HI and LARP generally achieve high PLQY, indicating reduced non-radiative recombination. However, the broad and overlapping PLQY ranges across different methods indicate that synthesis-dependent defect density and long-term stability cannot be reliably compared using PLQY alone (Figure 2). The PLQY values presented in Figure 2 are literature-reported values obtained under different sample and measurement conditions. Therefore, they should not be interpreted as a direct quantitative comparison of the intrinsic performance of the respective synthesis methods. Differences in concentration, substrate, surface treatment, encapsulation, and excitation conditions may contribute to the variation among the reported values. Because study-specific uncertainties are not consistently reported, uniform error bars are not shown. Additional characterization techniques, including carrier-lifetime measurements, defect-state analysis, and stability testing, are therefore required for a comprehensive assessment of material quality. These considerations highlight the need to examine the intrinsic and extrinsic degradation mechanisms that govern the stability of CsPbBr_3_, which are discussed in the following section.

## 3. Stability Challenges in HPs

The instability of HPs arises from the combined influence of intrinsic material properties and external environmental stressors that collectively affect their structural, optical, and electronic stability. Intrinsic degradation originates from the soft ionic lattice of perovskites, which facilitates ion migration, defect formation, and structural transformations [70,71,72]. The accumulation of mobile ionic species can generate local lattice strain and non-radiative recombination centers, ultimately reducing device performance and long-term stability. Synthesis conditions, crystal quality, and morphology further influence defect density and ionic mobility, thereby affecting degradation behavior. Beyond these intrinsic factors, moisture, oxygen, illumination, elevated temperature, and polar solvents can further destabilize the perovskite lattice through hydrolysis, thermal decomposition, photoinduced defect formation, and halide loss [25,26,27]. These environmental stressors often accelerate intrinsic degradation processes by promoting defect generation and enhancing ion migration. Hence, in this review, we primarily focus on the intrinsic factors that lead to HP degradation.

The dominant instability mechanisms in CsPbBr_3_ depend strongly on material form and length scale. Bulk crystals primarily provide insight into intrinsic lattice dynamics and point-defect energetics [21,29]. NCs are strongly influenced by surface coordination, ligand dynamics, and their high surface-to-volume ratio [73]. In polycrystalline films, grain boundaries and extended defects can provide additional pathways for ionic transport and defect accumulation [32]. Device architectures introduce interfaces, electric fields, charge injection, and illumination, which can influence ionic transport and degradation [74,75]. Accordingly, observations from these systems should be interpreted within their respective structural and operational environments rather than treated as directly interchangeable. In addition to material form and length scale, ionic transport in CsPbBr_3_ depends strongly on experimental and operational conditions. Illumination, temperature, electrical bias, moisture, oxygen, and the surrounding chemical environment can modify defect populations, surface reactions, and local electric fields, thereby influencing ionic transport and degradation [32,33,35,70,71,76]. Ionic transport under controlled conditions should therefore be distinguished from that observed under external stimuli or device-operating conditions. In this review, these external factors are considered primarily in terms of how they modify or accelerate the intrinsic instability processes of CsPbBr_3_.

In the following sections, the intrinsic and extrinsic degradation pathways are discussed individually to provide a detailed understanding of HP instability. The instability of CsPbBr_3_ can be understood through several fundamental lattice-related mechanisms. These include geometric constraints described by the Goldschmidt tolerance factor, anharmonic lattice dynamics, ionic migration in the soft perovskite framework, and defect formation at surfaces and interfaces. Together, these mechanisms provide the underlying physical basis for many degradation pathways observed in CsPbBr_3_-based optoelectronic devices. For clarity, the following subsections discuss the major instability processes individually. The order of these subsections is intended only to organize the discussion and does not imply a fixed causal or temporal sequence. These processes are interconnected, and their relative contributions and interactions depend on the material form and environmental or operational conditions. The strength of evidence supporting the proposed instability processes varies considerably. Some processes have been directly demonstrated experimentally, whereas others are supported primarily by indirect observations or theoretical calculations. Throughout this review, these distinctions are considered when evaluating the proposed mechanisms, and Table 2 explicitly identifies the evidence type for ion-migration-related observations.

### 3.1. Goldschmidt Tolerance Factor (t)

The instability of CsPbBr_3_ can be partly explained by its Goldschmidt tolerance factor (*t*), which is widely used to assess lattice geometry, octahedral distortion, and structural stability in perovskites [75,81,82]. It is defined in terms of the ionic radii of the A-site cation (*r_A_*), the B-site cation (*r_B_*), and the X-site anion (*r_X_*) (Equation (1)), providing a measure of their geometric compatibility within the perovskite lattice [83]. For stable three-dimensional (3D) perovskite structures, the tolerance factor typically falls within the range of ~0.8–1.0 [75,81,82]. A Goldschmidt tolerance factor close to unity (*t* ≈ 1.0) is generally associated with an ideal cubic perovskite structure [84]. In contrast, lower tolerance factors (0.8 ≤ *t* < 0.9) favor octahedral tilting and structural distortion, resulting in tetragonal, orthorhombic, or rhombohedral crystal phases [85].

(1) $$t=\frac{\left(r_{A}+r_{X}\right)}{\sqrt{2}(r_{B}+r_{X})}$$

The tolerance factor of CsPbBr_3_ is relatively low, typically estimated at ~0.86 [82,86,87]. This value falls within the conventional stability range for perovskite formation. Nevertheless, because the reported tolerance factors remain below unity, CsPbBr_3_ favors octahedral tilting and orthorhombic distortion at room temperature rather than a perfectly cubic structure [67,88,89]. While this moderate “*t*” value explains the intrinsic phase stability of CsPbBr_3_ compared with hybrid organic–inorganic perovskites, it does not fully determine the dynamic lattice behavior of the material [22,88,89]. Instead, a tolerance factor below unity favors structural distortions and lattice flexibility. Such lattice softness lowers the energetic barrier for octahedral tilting, defect formation, and ionic displacement. Therefore, CsPbBr_3_ becomes more vulnerable to instability under external stress [29,72,89]. Experimental and theoretical studies indicate that halide vacancies in CsPbBr_3_ exhibit intrinsically low migration barriers in the order of ~0.1 eV in idealized bulk lattices [29]. This enables rapid ion migration under electric fields, illumination, or thermal activation, even when the crystal structure remains unchanged [29,73,88].

Moreover, lattice distortions at surfaces, grain boundaries, or strained regions locally disrupt the ideal perovskite geometry, creating favorable pathways for ion migration and defect accumulation [72,90]. As a result, increased trap density, non-radiative recombination, and electrical hysteresis develop, ultimately degrading optoelectronic performance and operational stability [72,90,91]. The Goldschmidt tolerance factor of CsPbBr_3_ supports its crystallographic formability and phase stability. However, this geometric parameter alone cannot ensure long-term operational stability, because the dynamically soft lattice facilitates migration and defect evolution [73,90,92].

### 3.2. Anharmonicity

**Figure 3:**
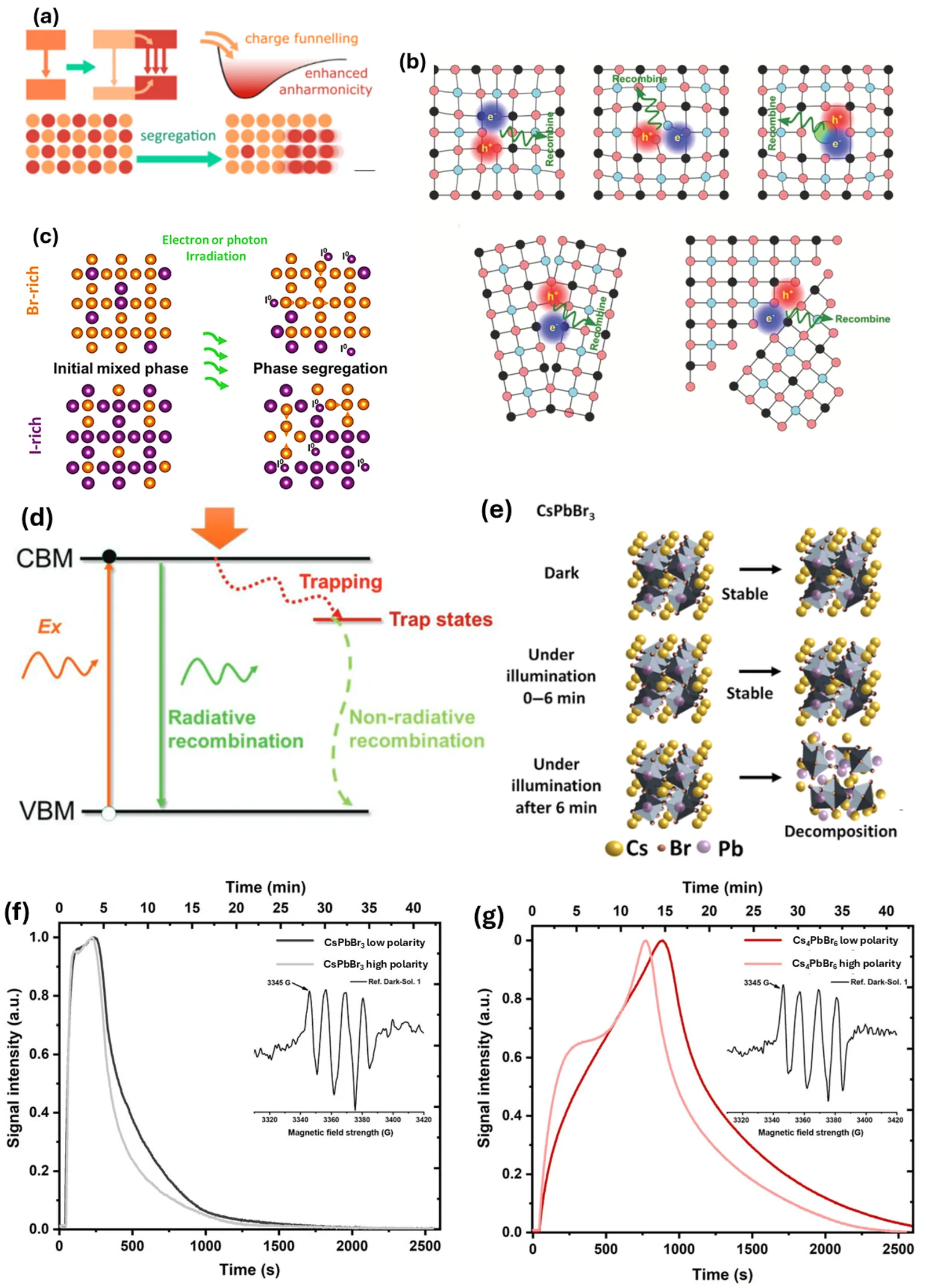
Schematic illustration of (**a**) halide segregation associated with lattice anharmonicity [106], (**b**) ion transport mediated by charged point defects [107], (**c**) light-induced phase segregation in mixed-HPs [108], (**d**) non-radiative recombination [109], (**e**) light-induced decomposition of CsPbBr_3_, and EPR kinetic spectra showing the decomposition of (**f**) CsPbBr_3_ and (**g**) Cs_4_PbBr_6_ NCs in solvents of different polarity [35]. All panels are reproduced with permission from the indicated references.

Anharmonic lattice dynamics are a key contributor to structural instability and operational degradation. The soft and highly polarizable Pb–Br framework undergoes large-amplitude vibrations and strong phonon–phonon interactions that deviate from harmonic behavior [93,94]. Low-energy vibrational modes associated with partially localized Cs^+^ oscillations and Br-ion motion induce dynamic distortions of the PbBr_6_ octahedra. These anharmonic vibrations lead to glass-like thermal transport, where strong phonon scattering suppresses conventional phonon-mediated heat conduction. As a result, CsPbBr_3_ exhibits very low thermal conductivity (~0.3–0.5 W m^−1^ K^−1^) and pronounced temperature-dependent lattice disorder [95,96]. First-principles molecular dynamics simulations based on density functional theory (DFT) with spin–orbit coupling have demonstrated that anharmonic lattice fluctuations strongly influence the electronic structure of CsPbBr_3_. These fluctuations increase the band gap by as much as ~450 meV in the cubic phase at 425 K relative to harmonic or static lattice descriptions [94]. They additionally enhance electron–phonon coupling through low-frequency Pb–Br modes (~20 meV) [97]. This enhanced coupling shortens carrier lifetimes and increases non-radiative recombination under illumination [94,98]. The soft and anharmonic lattice of CsPbBr_3_ facilitates halide migration, with Br-vacancy migration barriers typically ranging from ~0.2 to 0.4 eV [21,77]. This reduction enables transient diffusion pathways that promote Br^−^ transport, defect clustering, and phase segregation (Figure 3a). Multiphonon scattering associated with zone-edge lattice vibrations can induce transient local structural distortions, which may generate non-radiative recombination centers [31,93,99]. Importantly, the intrinsic structural characteristics associated with the favorable optoelectronic properties of CsPbBr_3_ are also closely connected to its instability. Lattice softness, dynamic structural fluctuations, and lattice polarizability are associated with important electronic and carrier–lattice properties [21,100,101]. However, the same soft and dynamic lattice can facilitate structural distortion and ionic transport [21,29]. Consequently, stabilization should not necessarily aim to completely suppress lattice dynamics or maximize lattice rigidity. Instead, an important challenge is to restrict detrimental ionic transport and degradation while preserving the favorable structural and electronic characteristics of CsPbBr_3_.

### 3.3. Soft Ionic Lattice and Ion Migration

The weak bonding in CsPbBr_3_ makes its lattice soft and easily decomposable. This mechanical softness reduces the elastic modulus of the framework and lowers the formation energy of halide vacancies. As a result, defects form easily, and a significant population of mobile ionic species exists even at room temperature. These defect-rich conditions directly promote ion migration, as Br^−^ and Cs^+^ ions diffuse through vacancy-mediated pathways within the lattice (Figure 3b). Experimentally determined activation energies are ~1 eV for Br^−^ and Cs^+^ in polycrystalline and device-relevant architectures [28,29,102]. Electrical measurements further reveal high mobile-ion densities (10^16^–10^18^ cm^−3^) and ionic mobilities in the order of 10^−10^–10^−8^ cm^2^ V^−1^ s^−1^ [75,102]. These transport properties allow ions to redistribute over sub-micrometer distances on millisecond-to-second timescales under operational electric fields or illumination [71,88]. Grain boundaries serve as energetically favorable pathways, especially for Br^−^ ions. As a result, ionic drift becomes faster in these regions, leading to localized defect accumulation, structural deformation, and the development of nonstoichiometric domains [32]. These ion-driven processes give rise to several major instability pathways in CsPbBr_3_. They contribute to the formation of non-radiative recombination centers, phase transitions, halide loss, and progressive chemical decomposition under prolonged bias, heat, or illumination [88,103,104]. However, the intrinsically soft lattice of CsPbBr_3_ remains a fundamental cause of structural and operational instability [81,105].

The energetic parameters reported for ionic transport in CsPbBr_3_ should be interpreted according to the material form, transport pathway, method, and experimental conditions. Calculated migration barriers and experimentally derived activation energies represent different physical quantities and should not be directly compared. Calculated migration barriers describe the energy associated with a specific microscopic ionic or vacancy migration pathway [21,29]. In contrast, experimentally derived activation energies describe effective thermally activated ionic transport and may include contributions from defect formation, trapping, grain boundaries, interfaces, and multiple transport pathways [29,74,77]. Therefore, differences among the reported values do not necessarily indicate disagreement between studies but may reflect differences in the physical quantities measured or calculated, as well as differences in material form, transport pathway, and measurement conditions. The reported energetic parameters associated with ionic transport in CsPbBr_3_ are summarized in Table 2, with a distinction between calculated microscopic migration barriers and experimentally derived activation energies.

Compositional engineering can regulate ion migration by modifying lattice geometry, bonding, and defect energetics. A-site substitution, such as partial replacement of Cs^+^ by Rb^+^, can alter the lattice dimensions and Goldschmidt tolerance factor [110]. B-site substitution provides stronger evidence of control over ionic transport, with selected dopants reported to increase defect formation energies and halide migration barriers [81,111]. X-site substitution also modifies the local migration landscape, although its effect on migration barriers is strongly composition-dependent [112]. Overall, the effectiveness of compositional engineering in regulating ionic transport depends on the substituted site, dopant identity, composition, and local lattice environment [81].

Ion migration and degradation in CsPbBr_3_ are strongly influenced by environmental and operational conditions. Illumination and applied electric fields can modify ionic transport, while humidity and atmospheric composition strongly affect degradation behavior (Table 2). However, direct quantitative comparisons of ion migration across illumination, humidity, and bias conditions remain limited. In particular, humidity-dependent studies generally evaluate optical or structural degradation rather than directly measuring ionic transport. This lack of directly comparable measurements represents an important knowledge gap in understanding condition-dependent ion migration in CsPbBr_3_.

### 3.4. Defect Formation and Surface Instability

CsPbBr_3_ NCs exhibit a defect landscape that differs substantially from that of bulk crystals because of their high surface-to-volume ratio and surface-dependent chemistry. Reported bulk perovskite defect densities are typically in the order of ~10^11^–10^16^ cm^−3^. As discussed by Vale et al. [73], applying these bulk defect densities to a ~10 nm perovskite NC would correspond to approximately one bulk-type defect per 10^2^–10^7^ NCs. This estimate does not represent the total defect density of NCs. Bulk defect densities and surface-related defect densities cannot be directly compared because they are generally reported using different dimensional metrics. Therefore, bulk volumetric defect densities should not be used to quantitatively infer surface-trap densities in NCs. Surface vacancies, under-coordinated ions, ligand-related defects, and surface reconstruction can contribute substantially to carrier trapping and recombination because of the high surface-to-volume ratio and dynamic NC–ligand environment [113]. Consequently, elevated trap densities shorten carrier lifetimes from hundreds of nanoseconds (ns) to below 10 ns, suppress optical gain, and compromise optoelectronic performance. Stoichiometry engineering has been demonstrated to suppress self-doping and reduce defect populations by more than an order of magnitude [92,114]. Surface and interfacial degradation is further amplified by ligand desorption during aging, which exposes under-coordinated Pb sites and promotes halide loss.

Therefore, ligand chemistry and interface passivation play a key role in determining the stability of NCs. Native oleic acid (OA) and oleylamine (OAm) ligands typically limit PLQY to ~40–60%. In contrast, hybrid or multidentate ligand reconstructions suppress interfacial trap states and increase PLQY to 90–95% [51,52,60]. Beyond ligand identity, the surrounding chemical environment also governs how quickly these surface trap states are generated once the NCs are removed from their synthesis matrix. Environmental stressors such as oxygen, moisture, and illumination accelerate interfacial degradation, often causing >80% PL loss within hours in unprotected films. Encapsulation strategies can limit this loss to ~13–15% over comparable timescales [80]. A related but distinct vulnerability arises from the acid–base chemistry of the ligand-passivated surface itself, independent of ambient humidity or light exposure. Acid–base reactivity plays a major role; strong acids (pK_a_ ≈ −1 to −2) can severely degrade NCs, whereas weaker acids primarily displace surface ligands. Halide-rich post-treatments can reverse vacancy formation, extend PL lifetimes, and restore emission severalfold [53]. Collectively, these findings indicate that controlling surface defects, ligand coordination, and environmental exposure is essential for stabilizing the intrinsically reactive surfaces of CsPbBr_3_ NCs.

### 3.5. Phase Segregation

Although phase segregation is absent in pure CsPbBr_3_, it provides insight into how ion migration and defect dynamics create additional instability in mixed-HPs. Phase segregation is the externally induced separation of mixed-HPs into compositionally distinct halide-rich phases. This phenomenon does not occur in pure CsPbBr_3_, which contains only a single halide species. In mixed-halide CsPb(Br_x_I_1−x_)_3_ NCs, this process is governed by ion migration, local stoichiometry fluctuations, and structural instability under illumination or thermal/electric bias [115]. A key quantitative trend is the strong size dependence: NCs with side lengths ≤22 nm show no resolvable Br-rich or I-rich domains. In contrast, larger particles exhibit clear multiphase contrast and PL shifts of several tens of nm toward the bandgap of the dominant halide phase (Figure 3c) [108,116]. Even below this threshold, minor spectral changes such as a ~3 nm PL blue-shift can occur due to subtle compositional rearrangements rather than full phase separation [108]. Photoinduced segregation processes can further drive mixed-halide NCs toward Br-rich configurations. Under continuous illumination, PL can evolve to ~520 nm within approximately 80 min, reflecting light-driven halide migration and lattice reconstruction [117].

These behaviors are enabled by relatively low ionic-migration activation energies in CsPbBr_3_-based materials, which support vacancy-assisted ion motion under modest stimuli [74]. Such ionic mobility contributes to structural relaxation, defect accumulation, and irreversible PL degradation over timescales of tens of minutes to hours. Together, these quantitative trends demonstrate that phase segregation is strongly dependent on both NC size and applied electric field. This behavior has critical consequences for the long-term optical and structural stability of mixed-HPs NCs.

### 3.6. Non-Radiative Recombination

Non-radiative recombination in CsPbBr_3_ is primarily governed by electronically active defects that introduce mid-gap trap states and offer ultrafast carrier-capture pathways. In NCs, the overall non-radiative decay rate typically ranges from 10^7^ to 10^8^ s^−1^ for moderately passivated systems. This rate can be comparable to or exceed the radiative recombination rate under ambient conditions. The magnitude of this non-radiative channel is directly tied to the material’s trap density, which varies substantially with sample form. Trap densities (N_t_) in well-prepared microcrystals or single crystals commonly lie near 10^14^–10^15^ cm^−3^, with reported values around N_t_ ≈ 3.8 × 10^14^ cm^−3^. In contrast, colloidal NCs often exhibit N_t_ ~10^16^ cm^−3^ due to their large surface-to-volume ratio [42,52]. These static trap-density differences are reflected dynamically in time-resolved PL measurements. Time-resolved optical studies reveal multiple decay pathways, including sub-nanosecond electron-trap capture processes (0.3–0.8 ns, corresponding to ~1–3 × 10^9^ s^−1^) as well as surface-mediated recombination channels occurring over several nanoseconds (2–5 ns, ~2–5 × 10^8^ s^−1^). Both mechanisms effectively compete with direct band-edge radiative recombination, which typically occurs on ~10 ns timescales. However, the overall PL lifetime can extend to several tens of nanoseconds because of trapping, detrapping, and delayed-emission processes [113,118].

The relative contributions of radiative and non-radiative pathways can be quantified using PLQY-derived rate constants. For example, CsPbBr_3_ perovskite quantum dots (PQDs) with an average lifetime of 43.2 ns exhibit a non-radiative recombination rate constant (*k_nr_*) of approximately 25.37 × 10^6^ s^−1^. The corresponding radiative recombination rate constant (*k_r_*) is approximately 15.95 × 10^6^ s^−1^ [119]. These comparable rate constants indicate that trap-assisted recombination contributes nearly half of the total carrier recombination. This trap-assisted contribution is not fixed, but is thermally activated, with recombination processes often exhibiting activation energies in the 0.1–0.6 eV range. Consequently, non-radiative activity increases under illumination or elevated temperature (Figure 3d) [109,120,121]. Beyond temperature and illumination alone, the chemical environment adds a further, cumulative source of new trap states. Environmental factors such as moisture, oxygen, and electric-field-driven chemical changes generate new surface defects and vacancy sites. These processes amplify performance losses and promote secondary transformations, including CsPb_2_Br_5_ formation and local Pb–Br network disorder. As a result, non-radiative recombination acts as both a diagnostic signature and an accelerator of performance degradation in CsPbBr_3_ NC emitters and devices [122,123,124].

### 3.7. Decomposition Under Harsh Conditions

CsPbBr_3_ degradation under harsh environments is primarily associated with phase transformation into CsPb_2_Br_5_, CsBr, and PbBr_2_. This process is strongly accelerated by the combined effects of humidity, heat, and illumination. Intrinsic thermal decomposition of NCs typically begins only at temperatures above ~300–400 °C under inert conditions. However, substantial optical and structural degradation can occur at much lower temperatures. This degradation is greatly accelerated in the presence of moisture or reactive gases [125,126,127]. Moisture-driven degradation begins with the adsorption and partial intercalation of H_2_O molecules into the lattice. These water molecules coordinate with Pb^2+^ centers, weaken the PbBr_6_ framework, and promote the conversion to CsPb_2_Br_5_ and soluble CsBr. Reactive gases such as H_2_S preferentially attack the surface, yielding CsBr and PbS while locally acidifying the environment, thereby further destabilizing the perovskite. Encapsulation with dense protective matrices can effectively suppress these degradation pathways. As a result, the CsPbBr_3_ structure can remain intact even under humid and elevated-temperature conditions [126,128].

Thermal stress, illumination, and humidity often act synergistically, producing faster degradation than any single stress factor. Under typical device operating conditions (60–100 °C), degradation is driven mainly by phase transformations, ion redistribution, and defect accumulation rather than by complete thermal decomposition. These processes reduce PL and increase non-radiative recombination. Sample architecture also governs the temperature at which decomposition sets in: well-crystallized bulk CsPbBr_3_ retains stable optical properties up to ~250–300 °C. At higher temperatures, structural degradation becomes significant. NCs are even more susceptible because ligand desorption and surface instability promote mass loss and decomposition as temperatures approach ~400 °C [31,129,130]. In contrast to these purely thermal pathways, light exposure introduces a photochemically driven degradation route. Continuous illumination can induce NC fusion, Ostwald ripening, and phase transformation of the perovskite lattice. Illumination in polar environments promotes the formation of ·OOH radicals, followed by Br^−^, Cs^−^, and Pb^−^ related radical species, marking the onset of lattice degradation. Radical-driven degradation begins within ~3–6 min for CsPbBr_3_ NCs (Figure 3e,f), compared to ~18 min for Cs_4_PbBr_6_ (Figure 3g), indicating lower intrinsic stability of the CsPbBr_3_ phase. Progressive radical accumulation under continued light exposure renders the degradation process self-sustaining, particularly in polar media [35]. In addition, combining exposure to light, heat, and humidity leads to rapid PL quenching in unprotected films. In contrast, optimized composite or encapsulated structures can retain more than 80–90% of their initial PL intensity after hundreds of hours under combined stresses. Reversible transitions between CsPbBr_3_ and CsPb_2_Br_5_ play a central role in this degradation process. Stabilizing matrices that immobilize ions and prevent water penetration can suppress this cycle, thereby improving long-term structural and optical stability [80,130].

### 3.8. Interplay Between Instability Mechanisms

Although the degradation mechanisms discussed above are often considered independently, the instability of CsPbBr_3_ arises from their strong interdependence. Lattice distortion, anharmonicity, ion migration, defect evolution, and chemical degradation are closely interconnected and collectively govern the long-term stability of HPs. The origin of this behavior can be traced to the relatively low Goldschmidt tolerance factor of CsPbBr_3_, which promotes octahedral tilting and lattice flexibility. This structural softness gives rise to anharmonic lattice vibrations that continuously alter the PbBr_6_ framework [100,101,131]. Under thermal, optical, or electrical excitation, these dynamic distortions lower the barriers for ionic transport and facilitate the formation and migration of defects [131,132]. The resulting halide vacancies and other point defects provide pathways for ion migration, particularly at surfaces, grain boundaries, and strained regions [29,132]. Ion redistribution subsequently generates local compositional fluctuations and additional defect sites, establishing a self-reinforcing degradation cycle [29,32,70,72,88,132]. As defect density increases, trap states are introduced within the bandgap, facilitating non-radiative recombination and reducing carrier lifetimes, PL efficiency, and device performance.

External stressors such as illumination, heat, moisture, and electric fields further accelerate these processes. Enhanced ion migration drives halide redistribution and, in mixed-halide systems, phase segregation. Simultaneously, defect-rich regions act as initiation sites for chemical decomposition through moisture adsorption, halide loss, and radical-driven reactions. The resulting degradation products further disrupt the lattice and generate additional defects. Therefore, the instability of CsPbBr_3_ should be viewed as a coupled, condition-dependent network of interacting processes rather than as a single degradation pathway [29,100,101,131]. Understanding these relationships is essential for developing effective strategies to improve the long-term stability of CsPbBr_3_-based materials and devices.

**Figure 4:**
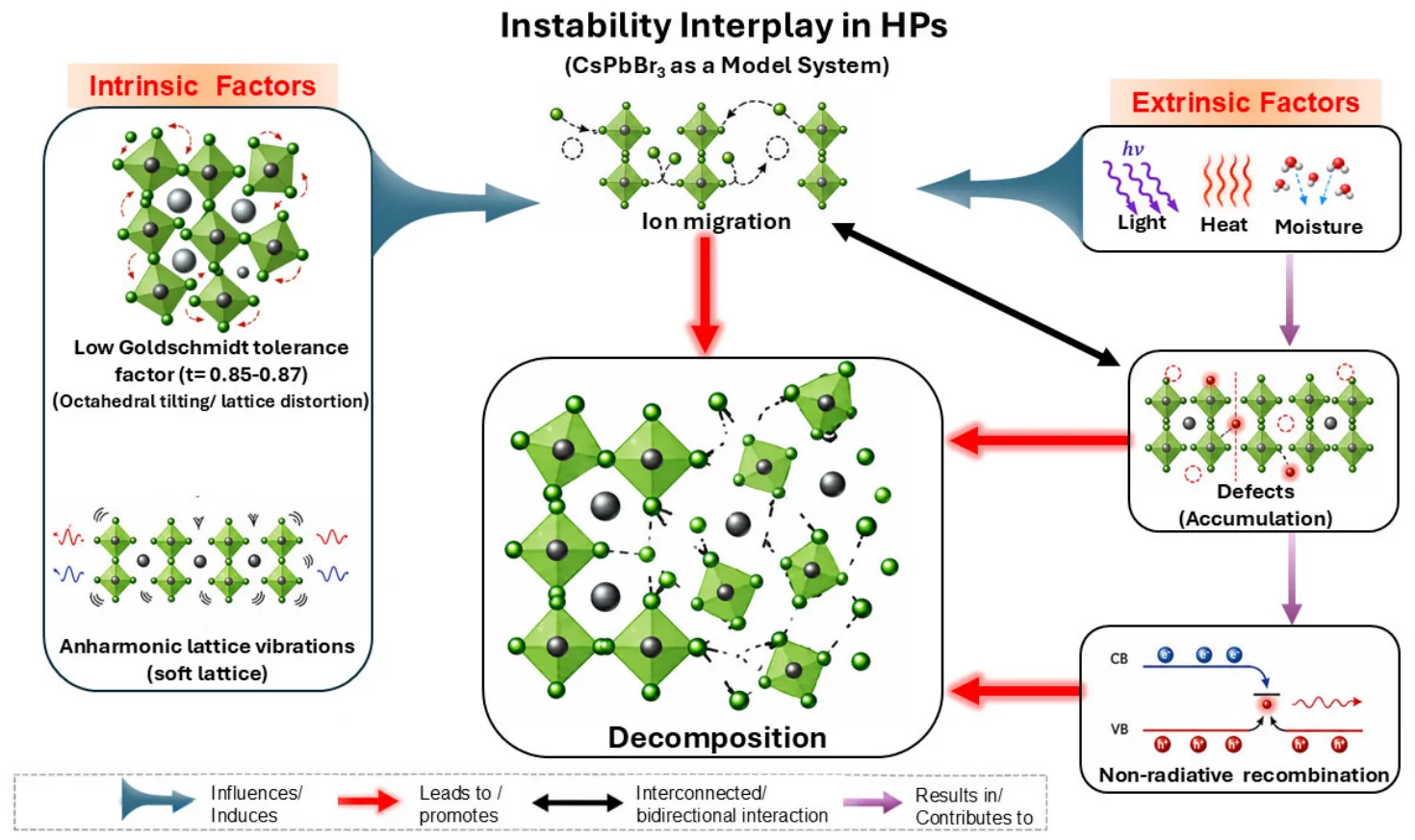
Schematic representation of the interplay among intrinsic and extrinsic factors and the coupled degradation processes in CsPbBr_3_. The arrows indicate interactions among these processes and do not represent a universal causal or temporal sequence.

The degradation processes illustrated in Figure 4 should not be interpreted as a universal sequential pathway. Rather, lattice dynamics, ion migration, defect evolution, carrier recombination, and chemical degradation are strongly coupled, and their relative contributions depend on the material form and environmental or operational conditions [29,70,71,72]. These processes can also interact through feedback mechanisms. For example, defects can facilitate ionic transport, while ion migration can generate or redistribute defects [29,32,71]. Chemical degradation can further introduce additional surface and bulk defect states, which may influence subsequent ionic transport and carrier recombination [70,71,72]. Therefore, the dominant processes and their temporal order may vary among bulk crystals, NCs, polycrystalline films, and device architectures.

### 3.9. Stability–Performance Trade-Off

The relationship between stability and optoelectronic performance presents an important challenge in HPs. The soft and polarizable lattice contributes to strong electron–phonon coupling and defect tolerance, but also facilitates ionic motion and structural instability. Therefore, simply increasing lattice rigidity may improve stability while altering some of the properties associated with the dynamic lattice. However, suppression of ion migration does not necessarily require sacrificing optoelectronic performance. Strategies that reduce mobile defects or restrict migration pathways, including defect passivation, compositional engineering, and interface modification, can improve stability while maintaining or even enhancing carrier and optical properties. Similar benefits of targeted surface passivation have been demonstrated in other HP compositions, where controlled PbI_2_ surface passivation reduced non-radiative recombination and improved device performance [133]. For example, surface passivation of CsPbBr_3_ NCs has been shown to suppress halide migration while simultaneously increasing PLQY and improving LED performance [134]. This demonstrates that ion migration can be suppressed without necessarily reducing PLQY or device performance. Nevertheless, the extent to which beneficial lattice dynamics can be separated from detrimental ionic transport remains insufficiently understood and requires systematic investigation.

## 4. Conclusions

The instability of CsPbBr_3_ arises from the interplay among synthesis-dependent structural features, intrinsic lattice properties, and external environmental stressors, rather than from a single degradation mechanism. Synthetic approaches strongly influence morphology, crystal quality, ligand coordination, and defect populations, thereby establishing the initial conditions that govern long-term stability. Although high PLQYs generally indicate effective defect passivation, PLQY alone cannot reliably predict operational durability and must be complemented by direct defect and stability analyses. The Goldschmidt tolerance factor (t ≈ 0.86) reflects the geometric tendency of CsPbBr_3_ toward octahedral distortion, while strong lattice anharmonicity contributes to its dynamic lattice behavior. This soft and dynamic lattice can facilitate ionic transport, although the reported energetic parameters depend strongly on the material form, migration pathway, method, and experimental conditions. In NCs, high surface-to-volume ratios and dynamic ligand binding further accelerate instability by exposing under-coordinated Pb sites and enhancing halide loss. Extrinsic stressors, including moisture, oxygen, illumination, elevated temperature, electric fields, and polar solvents, amplify these intrinsic vulnerabilities through hydrolysis, photoinduced defect formation, ion redistribution, and chemical decomposition. These processes are strongly coupled, and their relative importance and temporal order depend on the material form and environmental or operational conditions.

Future stabilization strategies should target multiple aspects of the coupled instability of CsPbBr_3_. Compositional engineering can regulate lattice structure, defect energetics, and ionic transport [81,111], while defect passivation and vacancy filling can reduce mobile defects and non-radiative recombination [70,135,136]. For NCs, improved ligand coordination and surface passivation can reduce surface defects and ligand loss [70]. Interfacial engineering and protective barriers can further limit ion redistribution and exposure to moisture, oxygen, and polar environments [70]. Stability strategies should also consider material form and operational conditions, as the dominant degradation processes may differ among NCs, films, bulk crystals, and devices. Several fundamental questions remain unresolved, particularly how lattice dynamics, defect evolution, and ionic transport interact under realistic operating conditions. Future approaches should therefore combine complementary stabilization strategies while preserving the favorable optoelectronic properties of CsPbBr_3_. Such an integrated approach will be important for enabling the reliable implementation of CsPbBr_3_ in next-generation photonic and electronic technologies.

## Figures and Tables

**Table 1:** The comparative results from the literature on the synthesis of CsPbBr_3_ and its characteristics.

HP	Synthesis Method	Absorption λ_max_ (nm)	Emission λ_max_ (nm)	PLQY (%)	Particle Size (nm)	Ref. No.
CsPbX_3_ (Cl/Br/I) NCs	HI	Tunable 410–700	Tunable 410–700 (CsPbBr_3_ ≈ 510–520)	Up to ~90%	4–15	[60]
CsPbBr_3_ NCs	HI	~480–520	~510–520	22–87%	1.8–13	[45]
CsPbX_3_ Nanoplates	HI	Thickness-dependent	Thickness-dependent (green/blue)	~84%	Nanocube = 8–10 Nanoplates = 20	[61]
CsPbBr_3_ NCs (size-dependent)	HI	Size-dependent	Size-dependent (green for larger dots)	30–60%	3.8–12.8	[62]
CsPbBr_3_ NCs	HI	~480–520	512–520	75%		[63]
CsPbX_3_	LARP	~500	380 to 693	80%	4.5 ± 0.9	[64]
CsPbBr_3_	LARP	516	~510–520	70–80%	8.4–13.5	[65]
CsPbX_3_	LARP	--	440–682	50–85%	7.8	[66]
CsPbX_3_ nanocubes	Solvothermal	510–520	410 to 700	Up to 80%	Nanocube = 14.7 Nanowire = 2.6	[67]
CsPbBr_3_ NCs	Emulsion Synthesis	--	--	75%	13.2 ± 1.9	[68]
CsPbX_3_	Microwave synthesis	--	410–692	10–92%	10–13	[50]
CsPbBr_3_	Mechanochemical ball-milling	--	~525	--	sub-100 > 1 µm	[51]
CsPbBr_3_/Cs_4_PbBr_6_ Heterostructure	Ball milling	--	--	66%	-	[52]
CsPbBr_3_ NCs (bulk scale)	Surfactant-assisted mechanochemical	Green region	--	7–88%	3.5–36.8	[69]
CsPbBr_3_ in mesoporous SiO_2_	Low-temperature molten-salt synthesis	--	--	89%	-	[58]
CsPbBr_3_	Molten-salt synthesis	513	523	20%	5.8	[59]

**Table 2:** Reported energetic parameters and condition-dependent ion-migration and degradation behavior in CsPbBr_3_ based on theoretical and experimental studies.

Material Form	Species/Defect	Method	Conditions	Result	Implication	Evidence Type	Ref.
Bulk CsPbBr_3_	Ionic transport	AC conductivity	Temperature-dependent	~0.25 eV	Effective activation energy	Experimental	[77]
CsPbBr_3_ single crystal	Ionic transport	Temperature-dependent transient current	Applied bias; variable temperature	0.160 ± 0.015 eV	Br-vacancy-mediated transport	Experimental	[29]
Orthorhombic CsPbBr_3_	Br− vacancy	DFT/NEB	Ideal bulk lattice	0.51 eV	Br-vacancy migration barrier	Theoretical	[29]
Orthorhombic CsPbBr_3_	Cs vacancy	DFT/NEB	Ideal bulk lattice	0.80 eV	Cs-vacancy migration barrier	Theoretical	[29]
Orthorhombic CsPbBr_3_	Pb vacancy	DFT/NEB	Ideal bulk lattice	1.88 eV	Pb-vacancy migration barrier	Theoretical	[29]
Cubic CsPbBr_3_	Br vacancy	DFT	Ideal bulk lattice	~0.65 eV	Br-vacancy migration barrier	Theoretical	[21]
CsPbBr_3_ surface	Br vacancy	DFT	Different surface pathways	~0.26–0.40 eV	Lower surface migration barriers	Theoretical	[21]
CsPbBr_3_ solar-cell device	Br− ion transport	Electrical measurement	Device, variable temperature	~0.74 eV	Effective activation energy	Experimental	[74]
CsPbBr_3_ thin film	Ion migration	Electrical/PFM/KPFM	Dark vs. illuminated; DC/AC bias	DC decreased; AC increased under illumination	Light effect depends on bias mode	Experimental	[78]
CsPbBr_3_ single crystal/photodetector	Br-ion migration	Electrical measurement/DFT	External electric field/bias	Field-driven ion migration and degradation	Electric fields drive ion migration	Experimental/ theoretical	[26]
CsPbBr_3_ single crystal	Ion migration	Electrical measurement	Atmosphere-controlled EFG growth	0.378 eV; lower dark-current drift	Reduced defects suppress ion migration	Experimental	[79]
CsPbBr_3_ NC film	Environmental degradation	PL/structural analysis	Humidity; air/O_2_ vs. N_2_; illumination	RH/light degradation in air; suppressed in N_2_	Humidity and photoinduced degradation	Indirect experimental	[80]

Abbreviations: AC, alternating current; DC, direct current; DFT, density functional theory; EFG, edge-defined film-fed growth; KPFM, Kelvin probe force microscopy; NEB, nudged elastic band; PFM, piezoresponse force microscopy; RH, relative humidity.

## Data Availability

No new data were created or analyzed in this study. Data sharing is not applicable to this article.
